# General Practice as a career choice among undergraduate medical students in Greece

**DOI:** 10.1186/1472-6920-7-15

**Published:** 2007-06-01

**Authors:** Anargiros Mariolis, Constantinos Mihas, Alevizos Alevizos, Vasilis Gizlis, Theodoros Mariolis, Konstantinos Marayiannis, Yiannis Tountas, Christodoulos Stefanadis, Anastas Philalithis, George Creatsas

**Affiliations:** 1 Department of General Practice/Family Medicine, Health Centre of Vyronas, Athens, Greece; 2 Divisions of Hygiene and Epidemiology, Department of Social Medicine, School of Medicine, University of Athens, Greece; 3 Section of Preventive Cardiology, Department of Cardiology, Vice-president of Medical School, School of Medicine, University of Athens, Greece; 4 Division of Health Planning, Department of Social Medicine, School of Medicine, University of Crete, Greece; 5 2nd Department of Obstetrics and Gynaecology, Dean of School of Medicine, University of Athens, Greece

## Abstract

**Background:**

Although General Practice (GP) was recognized as a medical specialty in Greece in 1986, the number of GPs is insufficient to cover needs and only few medical graduates choose GP as a career option. In the present study we investigated the profile of medical students in terms of their decisions regarding specialization and the possible association of career choices different from GP with the status of undergraduate training regarding GP.

**Methods:**

The sample consisted of final year students in the Medical School of the University of Athens, Greece. Students filled in a self-reported questionnaire focusing on medical specialization, and GP in particular.

**Results:**

Response rate was 82.5% with 1021 questionnaires collected, out of 1237 eligible medical students. Only 44 out of the 1021 (4.3%) respondents stated that GP is -or could be- among their choices for specialty. The most popular medical specialty was General Surgery (10.9%), followed by Cardiology (9.6%), Endocrinology (8.7%) and Obstetrics-Gynaecology (8.3%). The most common criterion for choosing GP was the guaranteed employment on completion of the residency (54.6%) while a 56.6% of total respondents were positive to the introduction of GP/FM as a curriculum course during University studies.

**Conclusion:**

Despite the great needs, GP specialty is currently not a career option among undergraduate students of the greater Medical University in Greece and is still held in low esteem. A university department responsible for undergraduate teaching, promotion and research in GP (where not available) is essential; the status of undergraduate training in general practice/family medicine seems to be one of the most important factors that influence physician career choices regarding primary care specialties.

## Background

The necessity of implementing innovative and developmental changes in primary care is of great importance, since the need for effective and efficient primary health care services worldwide, is imperative. One of the key-issues in improving the future care services is the human resources sector, which in practical terms is actually reflected in the number of medical students who choose General Practice/Family Medicine (GP/FM) as a specialty. There is a progressive decline in the number of physicians who choose GP/FM as a specialty in most countries worldwide, and many countries have recently increased GP's annual wages in order to attract postgraduate students in their residency training programmes. One of the most important factors that influences and promotes medical students in choosing a primary care specialty seems to be the undergraduate training programme. In Greece where we conducted our survey, according to the national medical specialties legislation, medical graduates register for the chosen specialty area and wait for a post opening. Depending on the popularity of the chosen specialty, a graduate may spend several years on a waiting list before beginning his/her residency. Even though there is a large need for General Practitioners [1], most of the medical students choose careers in other medical areas, a trend that is evident by the long waiting lists for residency entrance in all other specialties. Since 1986, when GP/FM was established as an independent medical specialty, the attention of policy makers was focused on how to direct new medical graduates to choose GP as a career in order to provide primary health care for the country's rural, and recently urban, population [2]. In Greek National Health System, General Practice and Family Medicine are considered as one medical specialty. General Practitioners/Family Physicians are totally and exclusively responsible for practicing Primary Health Care. However, the focus of policy on improving the training of General Practitioners and on providing incentives to make the career attractive did not extend to encourage the creation of departments of GP/FM in medical schools and the inclusion of General Practice or Primary Health Care in the medical curriculum. As a result, out of seven medical schools in the country, only the Faculty of Medicine of the University of Crete appointed, since it was established in 1984, academic staff in the discipline of Family Medicine [3] and has included a four-week clerkship in Primary Health Care in the final year of studies since the first group of students graduated in 1990 [4,5]. It should be stressed that although there is an increasing need for General Practitioners worldwide, few medical students are choosing this medical specialty at least in North America while its popularity varies in the United Kingdom. For example, as evidenced by the Canadian Residency Matching Service (CaRMS) reports, in 1982, 40% of Canadian medical students made GP/FM their first choice for residency training [6-11]. In 1996, this proportion had fallen to 32% and by 2002 to 29%. A similar picture is described in the United States [12-14]. There, the proportion of the medical school graduates selecting GP/FM fell from a peak of 17.3% in 1997 to 9.2% in 2003. Although there is a notable decline in the proportions of General Practitioners in the above two countries, such percentages are considered very high for the present situation in Greece. Currently, the proportion of medical students in Greece choosing GP/FM for specialization is as low as a 3.1% [15]. The reasons why medical school graduates do not choose GP/FM in Greece have not being investigated yet. It is speculated that part of the problem is due to the fact that the Greek healthcare system follows the trend towards super-specialized practice, made necessary by the explosion of new knowledge in the field of bio-medical research and made attractive by its better 'market value' [16]. However, the extremely low interest exhibited by Greek medical students is considered worthy of further investigation. It should be noted that at the present time in Greece, General Practitioners are the only medical specialists who have a secure career since they are appointed, immediately after accreditation as a specialist, to a tenured post in the Health Centres of the National Health System in rural Greece, where only approximately 1200 such posts out of 2700 are filled by specialists in GP. No other medical specialty has similar benefits in Greece today. The purpose of this study was to investigate the profile of senior medical students in the largest medical school in Greece, the Medical School of the University of Athens (Athens accounts for almost half the country's population and for the half of students in medical schools nationwide), in terms of specialization issues, to explore the reasons why GP/FM is held in such a low status as a specialty among medical students in Greece, as well as to investigate on the possible relation between undergraduate training programmes and career choices regarding primary care specialties.

## Methods

### Subjects

The target group consisted of students from the Medical School in the University of Athens. All medical students who were in the sixth final year of their academic studies and all graduating students who had not successfully completed their studies during the predefined 6 years of the Athens' medical school curriculum were invited to answer a questionnaire, described in detail below.

### Survey instrument and procedure

The survey instrument was a purpose-made, self-reported questionnaire with both closed and open-ended questions. The questionnaires were delivered after acquiring verbal consent. Apart from completing basic personal socio-demographic data, students were asked whether they had already chosen the medical residency they would like to follow and if yes, which would it be. Age, gender, parents' profession were self reported. The area of origin was also self-reported. After collecting the data, the classification of area to urban, rural or semi-urban was based on the Greek National Census of 2001[17]. Students who had not chosen yet their area of medical residency could indicate which specialties were in their first three choices, in an open-ended fashion. The questionnaire also included a question regarding the reasons for choosing a medical specialty. Here students could choose only one of four predefined answers (salary, scientific interest, low time to wait and social and professional prestige) or write in their own reason (again only one). Other items of the questionnaire referred to the sources of information and factors influencing their choice of medical specialty and GP/FM in particular. Students were also asked about the potential problems they were feeling they might encounter during their residency, what they believe is the best method for selecting new residents (e.g. waiting lists, exams etc.), the health care structure they would like to work at after finishing the residency and finally whether or not they are feeling satisfied with the choice to study medicine. All questions were closed-ended and the participants were invited to select only one of the predefined options. A second part of the questionnaire was devoted to issues more specifically related to GP/FM. There were questions about whether they were aware of GP/FM as a medical specialty and if respondents would be interested in choosing GP/FM. They also had to indicate the reasons for not choosing GP/FM as their specialty and the main sources of information about specialization in GP/FM. Again, the answers were pre-defined and the students were invited to choose the one that fitted the best to their opinion. Students were also asked to choose one of four proposed definitions of GP/FM, of which only one was correct. The 4 definitions of General Practice the medical student was invited to choose were: a) General approach of Medicine with basic knowledge of the main Medical specialties, b) First aid to emergency medical situations in removed regions, c) Humanistic oriented approach of Medicine with aim to prevent, treat and rehabilitate and d) Primary care of usual medical problems and reference to the suitable expert. The 3^rd ^definition was considered correct. Finally, students were asked to estimate the number of General Practitioners needed currently in Greece, and express an opinion about the usefulness of including GP/FM in the undergraduate course. The University of Athens ethics committee has examined and approved the protocol of our study regarding Helsinki Declaration about ethical principles for medical research involving human subjects. The above questionnaire had been pre-tested on family medicine residents at the Health Centre of Vyronas, Athens. During the main survey in the Medical School of Athens, the researchers directly administered the questionnaire to all medical students participating in the September-November 2004 examination period. It was made clear that answering the questionnaire was optional and that the anonymity of respondents was guaranteed.

### Data analysis

Three independent evaluators scored students' answers to open-ended questions, by identifying core themes and assigning them to categories. Some answers were assigned to multiple themes.

Statistical analyses included Pearson γ^2 ^and Fisher exact statistics where appropriate for categorical variables. The Mann-Whitney U statistic was also used for comparison of non-normally distributed continuous data. Results were considered statistically significant if the two-tailed p-value was less than 0.05. Data were analyzed using STATA™ (Version 8.0, Stata Corporation, College station, TX 77845, 800-782-8272).

## Results

### Respondents' socio-demographic information

Response rate was 82.5% with 1021 questionnaires out of 1237 completed and returned. Respondents' and non-respondents' mean age and gender did not differ significantly (p > 0.05). Students coming from rural or semi-urban areas of Greece were more likely to respond to the survey than students from urban areas (58.2% versus 41.8%, respectively; p = 0.017). Mean age of the respondents was 25.5 years, ranging between 23 and 38 years. Seven hundred seventy nine (779) (76.3%) respondents came from major cities, whereas 242 (23.7%) came from rural or semi-urban areas of Greece. A considerable percentage of the respondents (31.4%) had a parent also working as a medical doctor. Only 44 out of the 1021 (4.3%) respondents stated that GP/FM is -or could be- among their choices for specialty. No significant differences were observed by area of origin (i.e. urban versus semi-urban/rural) between the above group of potential General Practitioners *(pGPs) *and the group of students who did not report GP/FM among their specialty choices *(non-pGPs) *(p > 0.05). However, the mean age differed significantly between the pGPs and the non-pGPs, with pGPs being the oldest ones (mean difference 3.6 years; p = 0.012). PGPs were found to be less literate regarding computer use compared to non-pGPs (40.9% vs. 70.3%, respectively, p < 0.1). The proportion reporting knowledge of a foreign language did not differ between the two groups (p = 0.333).

### Medical specialty issues

The proportion of medical students who reported having already chosen which specialty they would follow was 38.2%. The most popular medical specialty was General Surgery (10.9%), followed by Cardiology (9.6%), Endocrinology (8.7%) and Obstetrics-Gynaecology (8.3%). Only seven respondents (1.7%) had already chosen GP/FM as specialty. For those respondents who reported not being sure about their specialty, (61.8%), Internal Medicine was the most favourite among their first choices (15.9%), followed by General Surgery (11.2%), Cardiology (8.7%) and Neurology (7.4%). Only twelve of those respondents stated that they might choose General Practice as a first choice (1.9%). General Practice was also reported as a second choice for 8 of the undecided students (1.3%).

### General Practice specialty issues

The most common criterion for choosing a specialty among the pGPs was the guaranteed employment on completion of the residency (54.6%), whereas scientific interest in the chosen specialty seemed to be the major criterion for the group of non-PGs (60.7%). Better quality of life and lower chances for unemployment were two criteria that influenced the 9.8% and 6.5% of non-pGPs and the 11.4 and 54.5% of pGPs, respectively (p < 0.001) (Table 1). The main problem the respondents believed that they would confront during residency was dealing with bureaucratic issues for the pGPs (72.7%) and having to work hard for the non-pGPs (39.5%, p = 0.586). The majority of pGPs (79.5%) stated that the present system of entering a residency course should remain as it is, whereas a 70.1% of the non-pGPs would prefer an examination system rather than the present one for entering the residency (p = 0.179). Most of the pGPs (75.2%) and the non-pGPs reported preferring to settle in an urban setting (84.1%) (p = 0.181). Finally, a 18.2% of the pGPs and a 28.4% of the non-pGPs reported having already 'regretted' choosing medicine as a profession (p = 0.139). The pGPs estimated that the need for medical doctors in Greece is around 75,000, while the rest of the respondents made a more conservative estimation of around 30,000 to 50,000, (p = 0.322). Forty three out of 1021 respondents (4.2%) stated that they were not aware of GP/FM as a residency they could choose to follow in Greece (Table 2). The major obstacle discouraging respondents from choosing GP/FM (59.2%) as a specialty was reported to be the lack of specialization of this discipline, followed by perceived difficulty in finding a career post, low grade of acceptance by the medical community, and low social prestige (14.8%, 13.7% and 12.3% respectively). The majority of medical students (77.3%) stated that medical student experience on General Practice was either non-existent or inadequate. Both pGPs and non pGPs shared the same opinion (p = 0.074) (Table 3). The main source of information for GP/FM for the whole sample of students was their fellow students (76.9%), whereas the role of the University was rather limited (8.2%) (Table 4). Both groups shared similar opinions on this subject (p = 0.093). A 27.9% of the non pGPs has already regretted for his choice to follow a medical career, while a 18.2% of the pGPs had the same opinion (p = 0.157). Only 62.1% of the total sample of respondents knew the exact duration of the GP/FM residency; even a 27.3% of the pGPs gave a wrong answer to this question. A 65.2% of pGPs chose the right answer among the 4 proposed definitions for General Practice, while only 8.4% of the non pGPs answered correctly (p < 0.001). A significant 88.6% of pGPs were positive to the introduction of GP/FM as a curriculum course during University studies (Table 5), while 55.2% of the rest of medical students agreed to this (p < 0.001). It should be stressed that 71.2% of those students who were against the introduction of GP in the university curriculum also reported that they found information on this medical specialty non existent or inadequate (p = 0.031).

**Table 1:** Students' criteria for choosing medical specialty

	**Possible specialty choice group**		
**Criterion**	**non-pGPs**	**pGPs**	**Total**

	**N (Row %)**	**N (Row %)**	**N**

Short waiting time before admission for residency training program	82 (95.4)	4 (4.6)	86
Particular scientific interest and skills	593 (99.2)	5 (0.8)	598
High professional and social prestige	101 (97.1)	3 (2.9)	104
Higher overall income	42 (93.3)	3 (6.7)	45
Other	159 (84.6)	29 (15.4)	188
Total	977 (95.7)	44 (4.3)	1021

p < 0.001

**Table 2:** Level of awareness of GP/FM as a specialty among students

	**Possible specialty choice group**		
**GP/FM awareness**	**non-pGPs**	**pGPs**	**Total**

	**N (Row %)**	**N (Row %)**	**N**

No	43 (100)	0(0)	43
Yes	934 (95.5)	44 (4.5)	978
Total	977 (95.7)	44 (4.3)	1021

p = 0.253

**Table 3:** Students opinion on information provided about GP/FM in their University

	**Possible specialty choice group**		
	**non-pGPs**	**pGPs**	**Total**

	**N (Row %)**	**N (Row %)**	**N**

**Information provided**			

Non-existent	337 (94.9)	18 (5.1)	345
Inadequate	411 (94.7)	23 (5.3)	444
Satisfactory	176 (98.3)	3 (1.7)	179
Adequate	53 (100.0)	0(0)	53
Total	977 (95.7)	44 (4.3)	1021

p = 0.074

**Table 4:** Source of information about GP/FM

	**Possible specialty choice group**		
**Source**	**non-pGPs**	**pGPs**	**Total**

	**N (Row %)**	**N (Row %)**	**N**

Colleagues	754 (96.2)	30 (3.8)	784
University	81 (96.4)	3 (3.6)	84
Medical Journals	45 (91.8)	4 (8.2)	49
Internet	21 (87.5)	3 (12.5)	24
Parents/relatives	63 (96.9)	2 (3.1)	65
Other	13 (86.7)	2 (13.3)	15
Total	977 (95.7)	44 (4.3)	1021

p = 0.093

**Table 5:** Students' interest on the possibility of introduction of a GP/FM course on their University curriculum

	**Possible specialty group**		
**Would you be interested in the introduction of a GP/FM course?**	**non-pGPs**	**General Practitioners**	**Total**

	**N (Row %)**	**N (Row %)**	**N**

No	438 (98.9)	5 (1.1)	443
Yes	539 (93.3)	39 (6.8)	578
Total	977 (95.7)	44 (4.3)	1021

p < 0.001

## Discussion

The results of the present study were rather unexpected. The extremely low proportion of medical students reporting GP/FM among their specialty choices revealed a disappointing fact about the status of GP/FM in Greece. More precisely, only one student was determined to select GP/FM for his residency program, whereas only 5 more of the respondents were considering it as one of it's choices. This proportion is rather disappointing compared to "traditional" medical specialties, such as general surgery and internal medicine. Such low interest in GP/FM is significant since other studies have revealed that a student's initial career preference is an important predictor of what the student ultimately chooses as a career and that students tend not to switch into GP/FM if it was not being considered during the medical school [18,19]. Although General Practice was one of the major policy directions of the creation of the National Health System in the 1980's, and repeated efforts to introduce incentives for making this specialty attractive have been made, including, in 1997, almost automatic appointment to a tenured post in a Health Centre [20], GP/FM remains a choice of low priority in 2005. The medical model of health and illness still predominates, with GP/FM widely identified as 'inferior' to 'traditional' medicine (i.e. hospital and super-specialized medicine). The endurance of this model in Greece is no surprise since GP/FM has not received the needed support and promotion, 20 years after its recognition as a specialty. General Practice seems to be appreciated as an 'easy' medical specialty in Greece. Various factors have led to such an opinion, including political choices, fundamental differences in its residency program compared to any other medical specialty. All these reasons seem to shape an environment under which the medical students who perform poorly academically at the undergraduate level tend to be the ones to choose GP/FM as the easiest training choice. The above is also supported by the present results; students who identified GP/FM as one of their choices were less likely to be computer literate, more likely to be older and more likely (although not statistically significant) to be concerned about the ensured salary than the scientific interest of their profession, in contrast to the rest of the students who had selected a different specialty. Additionally, the pGPs reported not preferring an examination system for entering a residency, in contrast to the non-pGPs. If the percentage and quality of students interested in GP/FM is found to reflect the choice of GP/FM as a residency and a profession, serious problems may eventually arise for the National Health System, taking into consideration the new model that is to be applied soon and which emphasizes Primary Health Care. The lack of any kind of information provided for choosing a specialty, either GP/FM or another one, should be also highlighted. For instance, a 73.3% of nGPs and 83.3% of pGPs set the number of General Practitioners needed in Greece to less than 5000 instead of 8000, as it is estimated by recent studies [20-22]. As it is reported, the Athens Medical School has not managed to provide adequate career information services that could help students choose the right medical specialty, according to ability, interest, need and personality factors and eventually to inspire students towards GP/FM. And this, despite the fact that the Medical School of Athens has organised a series of career and speciality orientation seminars in the last couple of years. Furthermore, a new elective course inGeneral Practice will be offered as of the Academic year 2006–2007. Moreover, medical students are trained in GP/FM mainly from faculty coming from other medical specialties. This fact may have generated a 'hospitalized' way of medical thinking and by no means to the health promotion and disease prevention way of thinking that Primary Health Care services should centre upon. In conclusion we feel that based on the current study, systematic actions should be taken in medical schools in order to educate and recruit more medical students to GP. Such strategies could be university courses and programs about Primary Care and General Practice [23], spending more time in Community training rather than in hospitals, with General Practitioners, as educators [24,25] and exposure to the General Practitioners' working model and rural practice at an undergraduate training level [5,26-32]. It is vital for GP recruitment and continuous education to introduce and fortify this medical specialty in the undergraduate medical curriculum, since as already stressed, exposure to GP during early university training leads to increased interest in it as a career choice [21,22]. Particularly for Greece, it seems that the first experiments on GP/FM residency have dramatically failed and that the main way for the improvement of the status of GP is to establish a university department, as is the case with all other medical specialties in the Greek Universities. This department should educate medical students in GP issues, recruit and train GP residents, offer post-graduate studies and care for the improvement of all Primary Health Care services in the country. The department of Social Medicine of the University of Crete, including the Division of Social and Family Medicine, has taken several initiatives in promoting undergraduate and postgraduate education and training in GP and in encouraging research in GP and Primary Health Care and has contributed substantially to the development of an active network between the Health Centres on the island of Crete [33,34]. The present study has several limitations, besides the low response rate achieved, indicative of the students' low interest in the subject. The Medical School of Athens, even though it is the largest [35], most popular and thus the one with the most competitive entry requirements, cannot be fully representative, something that is also evident from the distribution of the origin of the respondents which does not reflect the total population [36]. It would be interesting to conduct similar studies in the other medical schools in Greece, with the additional aim of examining whether differences exist in the way that the medical students of the University of Crete view GP. Additionally, a large number of students participating to the study, although in their senior year, had not made their final choices in terms of their specialization. A follow-up after graduation and until finalization of their residency choice would shed further light on the matter and it is strongly recommended to be pursued in future research.

## Conclusion

The professional status of GP/FM seems to be in a state of continuous crisis in Greece and much more has to be done in order to enhance GP/FM and put it higher on the list of priorities for specialization in medical students.

## Competing interests

The author(s) declare that they have no competing interests.

## Authors' contributions

AM conceived of the study, participated in its design and coordination and revised the manuscript.

CM conducted the statistical analysis, participated in its design and coordination, and drafted the manuscript.

AA participated in the design of the study, acquisition of data, analysis and interpretation of data, and revised the manuscript.

VG participated in the design of the study, acquisition, analysis and interpretation of data.

TM participated in the design of the study, acquisition, analysis and interpretation of data.

KM participated in the design of the study, acquisition, analysis and interpretation of data.

YT conceived of the study, participated in its design and coordination and revised the manuscript.

CS conceived of the study, participated in its design and coordination and revised the manuscript.

AP conceived of the study, participated in its design and coordination and revised the manuscript.

GC conceived of the study, participated in its design and coordination and revised the manuscript.

All authors read and approved the final manuscript.

## Pre-publication history

The pre-publication history for this paper can be accessed here:


